# Computed tomography and magnetic resonance imaging findings of intraorbital granular cell tumor (Abrikossoff’s tumor): a case report

**DOI:** 10.1186/s13256-016-0896-5

**Published:** 2016-05-13

**Authors:** Wei-Hsin Yuan, Tai-Chi Lin, Jiing-Feng Lirng, Wan-You Guo, Fu-Pang Chang, Donald Ming-Tak Ho

**Affiliations:** 1 Division of Radiology, Taipei Municipal Gan-Dau Hospital (Managed by Taipei Veterans General Hospital), No.12, 225 Lane, Zhi-Sing Road, Taipei, Taiwan 11260; 2 Department of Radiology, Taipei Veterans General Hospital, No.201, Sec. 2, Shipai Rd., Beitou District, Taipei City, Taiwan 11217; 3 School of Medicine, National Yang Ming University, No.155, Sec.2, Linong Street, Taipei, 112 Taiwan; 4 Department of Ophthalmology, Taipei Veterans General Hospital, No.201, Sec. 2, Shipai Rd., Beitou District, Taipei City, Taiwan 11217; 5 Department of Pathology, Taipei Veterans General Hospital, No.201, Sec. 2, Shipai Rd., Beitou District, Taipei City, Taiwan 11217

**Keywords:** Computed tomography (CT), Magnetic resonance imaging (MRI), Orbit, Granular cell tumor

## Abstract

**Background:**

Granular cell tumors are rare neoplasms which can occur in any part of the body. Granular cell tumors of the orbit account for only 3 % of all granular cell tumor cases. Computed tomography and magnetic resonance imaging of the orbit have proven useful for diagnosing orbital tumors. However, the rarity of intraorbital granular cell tumors poses a significant diagnostic challenge for both clinicians and radiologists.

**Case presentation:**

We report a case of a 37-year-old Chinese woman with a rare intraocular granular cell tumor of her right eye presenting with diplopia, proptosis, and restriction of ocular movement. Preoperative orbital computed tomography and magnetic resonance imaging with contrast enhancement revealed an enhancing solid, ovoid, well-demarcated, retrobulbar nodule. In addition, magnetic resonance imaging features included an intraorbital tumor which was isointense relative to gray matter on T1-weighted imaging and hypointense on T2-weighted imaging. No diffusion restriction of water was noted on either axial diffusion-weighted images or apparent diffusion coefficient maps. Both computed tomography and magnetic resonance imaging features suggested an intraorbital hemangioma. However, postoperative pathology (together with immunohistochemistry) identified an intraorbital granular cell tumor.

**Conclusions:**

When intraorbital T2 hypointensity and free diffusion of water are observed on magnetic resonance imaging, a granular cell tumor should be included in the differential diagnosis of an intraocular tumor.

## Background

First described by Abrikossoff in 1926 as a myoblastoma [1], granular cell tumors (GCTs), are rare neoplasms. Although GCTs can occur in any body part, the orbits are rarely affected [1, 2]. Most GCTs are benign, yet malignant forms have been sporadically reported. Only 10 to 15 % of GCTs are multicentric [3]. On histology, GCT cells feature acidophilic granular cytoplasm packed with lysosomes [1–3].

Orbital computed tomography (CT) and magnetic resonance imaging (MRI) with multiplanar views are normally used to assess intraorbital tumors or pseudotumors [4, 5]. Intraorbital GCTs are rarely diagnosed before surgery due to the rarity of GCTs involving the orbits. We report a case with a rare intraorbital GCT presenting with diplopia, blurred vision, and exophthalmos, originally identified as an intraorbital hemangioma based on its imaging features.

## Case presentation

A 37-year-old Chinese woman presented with a 6-month history of progressive blurred vision and double vision with right eye (OD) photophobia. She had no personal or family history of malignancy. She also denied any surgical history or history of chronic disease. Corrected Snellen’s visual acuity was 6/6 in her OD and 6/4 in her left eye (OS). Intraocular pressures were 18 mmHg in her OD and 16 mmHg in her OS. Movement was normal in her OS but was mildly restricted in all directions in her OD. Both her eyes were orthophoric.

Exophthalmometry measured 12 mm in OS and 15 mm (with proptosis) in OD. Both her pupils were round and measured 4 mm in diameter. The light reflex was reactive in OS but sluggish in OD. Her visual field was within normal limits in her OS but there was cecocentral scotoma in her OD. Dilated examination of each fundus revealed bilaterally flat optic nerve discs with clear margins. Cellophane maculopathy was present in her OD.

**Fig. 1:**
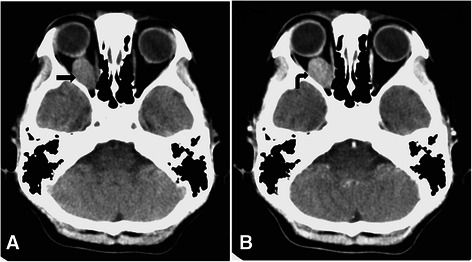
Intraorbital granular cell tumor on brain computed tomography. **a** Pre-contrast and **b** post-contrast axial brain computed tomography scans show a well-defined, ovoid, retrobulbar nodule (*arrow*) with slight contrast enhancement (*curved arrow*). The right globe is not indented by the tumor

**Fig. 2:**
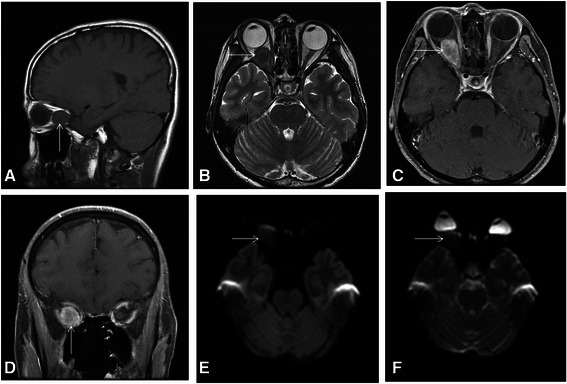
Intraorbital granular cell tumor on orbital magnetic resonance imaging. The tumor shows isointensity on **a** the sagittal T1-weighted image relative to gray matter and hypointensity on **b** the axial T2-weighted image. Diffuse heterogeneous enhancement with intravenous administration of gadolinium is noted on **c** axial and **d** coronal images. In **c** and **d**, the tumor appears in close association with inferior, lateral, and medial rectus muscles and the right optic nerve. The arrows are pointing to the tumor within panels **a** to **f**. On **e** diffusion-weighted image (*b* value = 1000) and **f** apparent diffusion coefficient map, the tumor shows isointensity relative to normal brain tissue without diffusion restriction

After ophthalmic tests, her clinicians arranged follow-up examinations within a month. Routine chest X-ray and laboratory results, including thyroid function tests, were normal. Pre-contrast and post-contrast brain CT revealed a solid, well-defined, ovoid, retrobulbar nodule measuring 15×15×27 mm within her right orbit with slight contrast enhancement. The globe was not indented and there was no bony erosion (Fig. 1a, b). On MRI of her brain and orbit, the intraorbital tumor presented with an isointense signal relative to gray matter on sagittal T1-weighted images (T1WIs) and axial fat-saturated T1WI, low signal intensity on axial T2-weighted images (T2WIs), and diffusely heterogeneous enhancement after intravenous gadolinium administration (Fig. 2a–d). There was no diffusion restriction of water on axial diffusion-weighted images (DWIs; *b* value = 1000) or apparent diffusion coefficient (ADC) maps, which revealed isointensity relative to normal brain tissue (Fig. 2e, f). On coronal post-contrast MRI, the tumor abutted inferior, lateral, and medial rectus muscles and her right optic nerve showed a flattened deformity. These findings were suggestive of an intraorbital hemangioma.

**Fig. 3:**
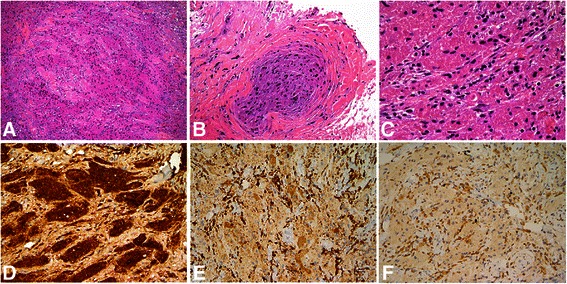
Pathologic specimens of intraorbital granular cell tumor. Histology shows **a** fibrotic soft tissue infiltrates with nests of polygonal tumor cells; **b** involvement of nerves around the tumor is also noted (hematoxylin and eosin stain, original magnification ×100); and **c** abundant eosinophilic granular cytoplasm and small nuclei. There is no cytologic atypia, increased mitotic activity, or necrosis (hematoxylin and eosin, ×200). Immunohistochemical stains for **d** S100, **e** CD68, and **f** MIB-1. The granular cells are diffusely positive for S100 (×400), and focally positive for CD68 (×200). The MIB-1 labeling index is 3, which represents low proliferation (×200)

Surgery was scheduled following the preoperative imaging diagnosis. The tumor was removed via right orbital-zygomatic craniotomy. Near total removal was achieved with some residual tumor attached to her optic nerve. The tumor measured 2.4×2.3×1.4 cm; it was firm, avascular, and gray-tan in color. Histology showed fibrotic soft tissue infiltrated with nests of polygonal tumor cells with abundant eosinophilic granular cytoplasm and small bland-looking nuclei. There was no cytologic atypia, increased mitotic activity, or necrosis. The tumor cells were poorly circumscribed and were noted in the cauterized resection margins. On immunohistochemical staining, the granular cells were immunoreactive for S100 and focally positive for CD68. The MIB-1 labeling index was 3, which represented low proliferation. These findings were consistent with a GCT (Fig. 3).

Six months after surgery, there was residual exophthalmos, and her eye movement and light reflex did not recover completely. Both her pupils were round and measured 4 mm in diameter in her OS and 5 mm in diameter in her OD. Intraocular pressures were 17 mmHg in both eyes. Visual acuity was 6/6.7 in her OD and 6/6 in her OS.

The first follow-up MRI at 6 months showed an ill-defined soft tissue component wrapping her optic nerve in the right retrobulbar region. The soft tissue around her right optic nerve revealed intermediate T1 and intermediate T2 signal intensity and contrast enhancement on MRI. Postoperative change (with residual tumor) was suspected. A follow-up MRI at 9 months showed regression of both contrast enhancement and size of the soft tissue component around her right optic nerve. Based on the results from the two follow-up MRIs, it was felt that the soft tissue remnant was compatible with postoperative change.

## Discussion

Ophthalmic GCTs can originate from the orbit (in only 3 % of all GCT cases), eyelids, optic nerve, extraocular muscles, lacrimal sac, ciliary body, conjunctiva, and caruncle [6]. The age of patients with reported ophthalmic GCT ranged from 3 to 74 years of age (average age, 40 years) without gender preference [6, 7]. In 84.6 % of orbital GCT cases, patients presented with progressive proptosis and diplopia developing over weeks to years [2].

Orbital GCTs tend to occur in the inferior half of the orbit. Diplopia results from involvement of extraocular rectus muscles, most commonly the inferior rectus muscle [2, 3, 6]. In the present case, coronal and axial CT and MRI showed a retrobulbar oval GCT with close association to the inferior, lateral, and medial rectus muscles and right optic nerve. Postoperative MRIs were performed to follow up the soft tissue remnant in the orbit. Multiplanar images assisted in selecting the route of intraorbital tumor extraction and in the postoperative detection of tumor removal with residual adhesion to her optic nerve or extraocular muscles.

To date, only 53 cases of GCT with ophthalmic involvement have been reported in the English literature [2, 7]. Only one of 53 cases was bilateral and another two cases were malignant tumors with distant metastases [2, 7]. Most intraorbital GCTs present as oval or round benign masses with well-circumscribed borders on CT or MRI [3, 6]. Few of the 53 cases have shown infiltration of the surrounding tissue [6]. Other typical CT findings include a mass which is isodense or slightly hyperdense relative to normal brain tissue with slight to strong enhancement after intravenous contrast administration, no calcifications, and no bony changes [2].

Orbital CT scans can better depict calcification and bony changes than MRI [8], but some benign intraorbital tumors, such as meningioma, schwannoma, and glioma, appear similar to GCTs on CT [9]. Another disadvantage of orbital CT compared with MRI is the radiation exposure during CT [8].

The present case showed characteristic features of GCT on MRI. Intraorbital GCTs are isointense to gray matter on T1WI, hypointense on T2WI, and show slight to strong contrast enhancement [10]. Unlike GCTs, intraorbital melanomas usually show hyperintensity on T1WI. Intraorbital cavernous hemangiomas, hemangiopericytomas, meningiomas, schwannomas, and gliomas show isointensity relative to gray matter on T1WI and isointensity or hyperintensity on T2WI [4]. Lymphomas, pseudotumors, and some metastatic lesions appear hypointense on T2WI reflecting a fibrotic nature, although morphology may vary [9, 10].

Intraorbital round or ovoid tumors (including GCT and metastases) usually present with variable contrast enhancement on CT or MRI [8–10]. Orbital cavernous hemangiomas show an enhancement pattern which is typically more heterogeneous than meningiomas, gliomas, schwannomas, or lymphomas [8, 9]. Therefore, T2-weighting hypointensity and homogenous enhancement on post-contrast CT and MRI are unusual for an orbital hemangioma.

Although no single ADC threshold can definitively differentiate benign from malignant orbital masses, malignant masses commonly show diffusion restriction with hyperintensity relative to normal brain parenchyma on DWI, hypointensity on ADC maps, and lower ADC values [11]. An ADC value <1.15×10^−3^ mm^2^/s is predictive of malignant orbital tumors with a sensitivity of 95 %, specificity of 91 %, and accuracy of 93 % [12]. Our case was suggestive of a benign intraorbital tumor as there was no diffusion restriction on DWI or ADC.

CT and MRI alone cannot differentiate intraorbital GCTs from other benign or malignant orbital tumors. However, GCTs are easily diagnosed based on histology because the tumor cells contain small vesicular nuclei and abundant granular eosinophilic cytoplasm on light microscopy [1, 4, 6]. GCT cells are also usually positive for S100 protein on immunohistochemistry. Myelin basic protein, vimentin, Leu 7, neuron-specific enolase, CD57, and CD68 may also be positive in GCTs [1, 4, 6]. GCTs may originate from various tissues including myoblasts, histiocytes, fibroblasts, and mesenchymal cells [2]. In our case, the strong expression of S100 protein supported a neural histogenesis. Currently, most investigators favor a Schwann cell origin [2, 4, 6].

The histopathological features of malignant GCTs include necrosis, hyperchromatic and pleomorphic nuclei, high nuclear to cytoplasmic ratio, vesicular nuclei with large nucleoli, absence of granules, and increased mitotic activity [1, 7]. Our case was compatible with a benign intraorbital GCT.

## Conclusions

Our patient presented with diplopia and was found to have a solitary, well-defined intraorbital mass adjacent to or within her extraocular muscles. There was no calcification within the mass on CT. MRI showed isointensity relative to gray matter on T1WI and hypointensity on T2WI with slight contrast enhancement. There was no diffusion restriction on DWI or ADC. When presented with these imaging features, GCTs should be included in the differential diagnosis of an intraorbital mass, particularly involving the inferior rectus muscle. Surgical biopsy is required to exclude a malignant lesion.

## Consent

Written informed consent was obtained from the patient for publication of this case report and any accompanying images. A copy of the written consent is available for review by the Editor-in-Chief of this journal.

## Abbreviations

ADC: apparent diffusion coefficient
CT: computed tomography
DWI: diffusion-weighted image
GCT: granular cell tumor
MRI: magnetic resonance imaging
OD: right eye
OS: left eye
T1WI: T1-weighted image
T2WI: T2-weighted image
